# Effects of dietary supplementation with an olive mill wastewater phenolic extract on the growth performance, oxidative status, and meat quality traits of finishing pigs

**DOI:** 10.3389/fvets.2026.1761378

**Published:** 2026-02-24

**Authors:** Flavia Ferlisi, David Ranucci, Raffaella Branciari, Katia Cappelli, Giuseppe Giglia, Luca Mechelli, Federica Mannelli, Samanta Mecocci, Gabriele Acuti, Ioannis Mourtzinos, Anastasia Kyriakoudi, Martina Crociati, Jiayong Tang, Erminio Trevisi, Massimo Trabalza-Marinucci

**Affiliations:** 1 Department of Veterinary Medicine, University of Perugia, Perugia, Italy; 2 Laboratory of Food Chemistry and Biochemistry, School of Agriculture, Aristotle University of Thessaloniki (AUTH), Thessaloniki, Greece; 3 Department of Agricultural Food, Environmental and Animal Sciences, University of Udine, Udine, Italy; 4 Animal Nutrition Institute, Sichuan Agricultural University, Chengdu, Sichuan, China; 5 Department of Animal Sciences, Food and Nutrition, Università Cattolica del Sacro Cuore, Piacenza, Italy

**Keywords:** antioxidant effect, diet, meat quality, olive waste, polyphenols, swine

## Abstract

Phenolic compounds from olive mill wastewater (OMWW) have a strong antioxidant capacity, so there is increasing interest in using them in feed for livestock, including pigs. This study tested the effects of dietary supplementation with a polyphenol extract from OMWW for female Landrace × Duroc heavy finishing pigs. There were three groups: the control diet (C group), the control diet supplemented with 74 ppm of OMWW polyphenols (P-LOW group), and the control diet supplemented with 225 ppm of OMWW polyphenols (P-HIGH group). Each experimental group comprised 45 pigs (*n* = 15 × 3 replicates), for a total of 135 pigs. The effects of the phenolic extract were assessed *in vivo* (growth performance) and postmortem (backfat thickness; pubertal status; histopathology of the liver, ovary, uterus, fat, and muscle; morphometry of the liver, ovary, and uterus; antioxidant status in the blood, muscle, and liver; effects on the quality and physicochemical characteristics of the raw meat). There were no significant differences between the treatments regarding the growth performance traits, histopathological and morphometric findings, and backfat thickness. However, there was an increase in 2,2-diphenyl-1-picrylhydrazyl (DPPH) radical scavenging activity in the liver of the P-HIGH group, alongside higher serum paraoxonase activity and ferric reducing antioxidant power. Meat quality analysis showed that cooking loss and redness (a*) decreased, while yellowness (b*) increased in the P-LOW and P-HIGH groups, indicating that OMWW polyphenols influenced the structure and water retention capacity of the meat. Additional research is required to better understand the role of dietary OMWW polyphenols in relation to the technological quality and antioxidant state of pork meat.

## 1 Introduction

The Mediterranean region is one of the world’s leading olive oil-producing areas. Italy produces approximately 6 million tons of olive oil per year, ranking second only to Spain (1). The olive oil industry generates several residues which are strong pollutants because of their high organic content and phytotoxicity. Thus, it is necessary to treat it appropriately (2). These wastes are also rich in bioactive molecules, including polyphenols (3, 4), which are secondary metabolites typically found in plants and known for their antioxidant, antimicrobial, anti-inflammatory, and immunomodulatory activities (5–10). Olive mill wastewater (OMWW) is a liquid by-product produced in high quantities from the olive oil industry. It contains a wide spectrum of water-soluble polyphenols, including hydroxytyrosol, tyrosol, oleuropein, verbascoside, phenolic acids, and flavones (3, 11, 12). In particular, hydroxytyrosol has very high radical scavenging activity because it contains several hydroxyl groups (13).

There are multiple issues related to animal feeding, including an increase in the demand and cost of raw ingredients. These issues raise concerns among producers regarding the long-term sustainability and economic viability of livestock production systems. This context has encouraged the use of unconventional natural feed additives derived from the agro-industrial sector, including by-products from the olive oil industry or extracts derived from these by-products (14, 15). The inclusion of these unconventional matrices in animal diets has been proven to reduce the environmental impact of by-products, contributing to the development of a circular economy (2, 16–18). Beyond these environmental advantages, recent studies have reported that the phenolic compounds contained in these products can help to improve animal health (including their oxidative status) and productive performance, and potentially reduce bacterial infections (7, 9, 19). Moreover, the supplementation of animal diets with olive polyphenols can contribute to maintain the oxidative stability (20–22) and lipid composition (23, 24) of meat. Recent studies showed that other natural phenolic sources, e.g., grapes, can enhance antioxidant activity in pigs when included in the diet (25, 26). To our knowledge, several studies have been conducted on the effects of olive oil by-products on the productive performance (22, 23, 27, 28) and meat quality (23, 29–31) of pigs but there has been almost no research on OMWW polyphenol extracts. Thus, the aim of this study was to investigate the effects of the dietary inclusion of an OMWW phenolic extract on the growth performance, morphological characteristics, backfat thickness, oxidative status, and meat quality of heavy pigs used to produce cured meat.

## 2 Materials and methods

### 2.1 OMWW extract

The OMWW extract was provided by (Stymon Natural products, P.C., Patras, Greece; www.stymon.com). The total phenol content (TPC) was measured with the Folin–Ciocalteu method according to Nenadis et al. (32), using a UV-1800 spectrophotometer (Shimadzu, Kyoto, Japan). The extract solution was prepared by mixing 0.5 g of OMWW extract with 25 mL of an 80/20 (v/v) mixture of methanol and water. Gallic acid was used as the reference standard, and the results are expressed as milligrams of gallic acid equivalents per kilogram (mg GAE/kg). The total phenolic content of the OMWW extract was 36.99 ± 58.9 mg GAE/kg. Hydroxytyrosol, tyrosol and oleuropein were detected in the extract at the following concentrations: 9.39 ± 17.4 g/kg, 1.09 ± 3.2 g/kg and 0.59 ± 0.4 g/kg through reversed-phase high- performance liquid chromatography (RP-HPLC-DAD) analysis.

#### 2.1.1 Analysis of polyphenols

Reverse-phase high-performance liquid chromatography with a diode array detector (RP-HPLC-DAD) was used to analyse the presence of the polyphenols hydroxytyrosol, tyrosol, and oleuropein in the OMWW extract, following the procedure described by Kyriakoudi et al. (33). It involved the use of a 1260 Infinity II Quaternary Pump VL with an autosampler, a 1260 Infinity II Diode Array Detector High Sensitivity, and an InfinityLab Poroshell 120 EC-C184μm column (150 × 4.6 mm inner diameter; Agilent Technologies, Santa Clara, CA, USA). The column temperature was set at 30 °C prior to analysis, the extract was filtered through a 0.45-μm PTFE filters (Frisenette, Knebel, Denmark) The compounds in the extract (an injection volume of 20 μL) were subjected to gradient elution using a mobile phase consisting of water (0.1% acetic acid) (A) and acetonitrile (B): 0 min, 5% (B), 0–10.0 min, 20% (B); 10.0–15.0 min, 30% (B); 15–18 min, 30% (B); 18.0–20.0 min, 50% (B); 20.0–21.0 min, and 100% (B); and 21–25 min, 5% (B). The total run time was 25.0 min, with a flow rate of 1.0 mL/min. Absorbance was measured in the range of 190–600 nm. The chromatographic data were processed using the OpenLab CDS version 3.5 software (2021, Agilent Technologies). The peaks were identified based on comparison of the retention times and spectral characteristics (absorption maxima) with the available standards.

### 2.2 Animal welfare and ethic statement

This study was approved by the Bioethics Committee of the University of Perugia (protocol number 399480) and performed under the ARRIVE guidelines. All animal care procedures followed the European recommendations (Directive 2010/63/EU) for the protection of animals used for scientific purposes and D.lgs 26/2014 (which implemented Directive 2010/63/UE in Italy). The pigs used in this study were raised and slaughtered for conventional meat commerce.

### 2.3 Animals and diet

This study was carried out on a conventional farm in a hilly area of Umbria, Italy. One hundred thirty-five female Landrace × Duroc finishing pigs (body weight: 102.7 ± 6.8 kg) were randomly assigned to three feeding groups (3 pens for each group and 15 pigs per pen) fed with the following experimental diets: (a) the control (C) diet, a commercial mash-form feed used in the finishing period (see Table 1 for the composition); (b) the C diet supplemented with 210 mg/day of polyphenols from OMWW (the P-LOW diet); or (c) the C diet supplemented with 630 mg/day of polyphenols from OMWW (the P-HIGH diet). The OMWW polyphenol extract was diluted in water (15 L of water per box) and mixed with the feed (2.8 kg/pig), to reach a final phenolic concentration of 74 ppm for the P-LOW diet and 225 ppm for the P-HIGH diet. The pigs were provided with an automatic wet feeding system and fed two times per day. Water was given *ad libitum*. All pigs underwent a 15-day adaptation period.

**Table 1:** Ingredients (% as fed basis) and chemical composition (g/100 g) of the diet.

Raw materials	% as fed basis
Grain corn flour	41.8
Grain barley flour	21.7
Wheat middlings	8.1
Proteins	2.5
Wheat flour middlings	9.0
Soybean meal	14.4
Mineral-vitamin supplement^1^	2.5
Analyzed nutrients	g/100 g
Moisture	11.20
CP^2^	15.10
Ether extracts	3.61
Ash	6.83
Crude fiber	4.03
NDF^3^	17.96
ADF^4^	5.79
ADL^5^	1.72
Starch	44.45
Ca	0.68
P	0.53

1 Mineral-vitamin supplement (mg or units/kg): 216,700 UI Vitamin A; 65,000 UI Vitamin D3; 4,000 mg Vitamin K3; 86 mg Vitamin K3; 50 mg Vitamin B1; 160 mg Vitamin B2; 450 mg calcium D-pantothenate; 100 mg Vitamin B6; 1 mg Vitamin B12; 800 mg Vitamin B3; 5 mg Biotin; 40 mg Vitamin B9; 15,000 mg Cholin chloride; 1.500 mg iron carbonate; 1,500 mg iron(II) sulfate monohydrate; 50 mg calcium iodate; 1,500 mg manganese oxide; 500 mg copper(II) sulfate pentahydrate; 8 mg sodium selenite; 3 mg selenomethionine hydroxylate; 1,000 mg zinc oxide; 13,000 methionin; 58,000 mg L-lysine monohydrochloride; 16,500 mg L-threonine;18,700 TXU endo-1,4-beta-xylanase; 16,000 FTU endo-1,4-beta-glucanase; 16,000 FTU 6-Phytase.

2 CP, crude protein.

3 NDF, neutral detergent fiber.

4 ADF, acid detergent fiber.

5 ADL, acid detergent lignin.

### 2.4 Feed analysis

The crude protein, crude fibre, ether extract, ash, calcium, phosphorous, moisture, and starch contents were assessed according to AOAC procedures (34). Neutral detergent fibre (NDF), acid detergent fibre (ADF), and acid detergent lignin (ADL) were analysed following published methods (35).

### 2.5 Pig performance measurements

The body weight (BW) of each pig was recorded at the beginning and end of the experiment. Average daily gain (ADG, g/pig/day) was calculated as the difference between the initial and the final BW divided by the duration of the experiment. The feed conversion ratio (FCR) was determined by dividing the daily feed intake by the daily gain. At the end of the experiment (day 85), the pigs were transported to a local authorised slaughterhouse and killed by bleeding after electrical stunning, according to the European Council Regulation (EC) No 1099/2009. After slaughtering, the carcasses were weighed to determine the hot carcass weight (HCW), and then stored in a chilling room (2 °C–4 °C) for 24 h. Finally, the yield at slaughter (dressing out percentage) was calculated as [(HCW/BW) × 100].

### 2.6 Postmortem analyses

At slaughter, 45 pigs (15 pigs per group, 5 pigs per pen) were randomly selected for postmortem evaluations. Livers, ovaries, and uteri were collected immediately following evisceration, whereas adipose and muscle samples were obtained at the end of the slaughter line, after the carcasses had been weighed but prior to chilling. Subsequently, the carcasses were stored at 4 °C for 24 h.

The backfat thickness (measured in centimetres) was assessed at the time of slaughter with an ultrasound scanner (RKU10, Kaixin Mansion, C-01, Economic Development Zone, Xuzhou, Jiangsu, China) equipped with a 4.0–6.5 MHz transducer. As described by Cisneros et al. (36), the transducer was set at 4.0 MHz and the depth of the scan was 10 cm. After lubrication with ultrasound gel, the transducer was placed on the right side of the carcass just below the last rib, in a vertical position. Three measurements were recorded for each carcass: one each at the upper, lower, and central portions of the scanned area. Then, the average backfat thickness was calculated.

#### 2.6.1 Histopathological and morphometric evaluations

The livers, ovaries, and uteruses were subjected to macroscopic analysis. In addition, liver, ovary, uterus, fat, and skeletal muscle samples were fixed in 10% buffered formalin for 24 h for histological analysis. Formalin-fixed and paraffin embedded (FFPE) samples were processed according to standard protocols, and 3-μm tissue sections were obtained and stained with haematoxylin and eosin. A scoring system was used to assess tissue changes, following recommendations reported by Gibson-Corley et al. (37). Specifically, the severity of leukocyte infiltration; hepatocellular degeneration and necrosis; hepatic fibrosis; fat atrophy and necrosis; muscle atrophy, hypertrophy, and regeneration; epithelial degeneration; epithelial necrosis; mucosal glandular hyperplasia; and lamina propria fibrosis of the uterus were scored from 0 to 3, based on the percentage of affected tissue: 0 = no tissue affected, 1 = less than 20%, 2 = 21–60%, and 3 = more than 61%.

For the reproductive tract, the presence of luteal bodies in the ovary and the presence of adenomyosis in the uterus were recorded as binomial factors (i.e., yes or no). The pubertal status of gilts was assessed macroscopically, as described by Vela et al. (38). The dimensions, features, and relevant abnormalities of the vagina, uterus (e.g., the uterine horns) and ovary were determined. The presence of follicles larger than 6 mm together with corpora lutea and/or corpora albicans were considered indicative of puberty.

#### 2.6.2 Oxidative status in the blood and tissues

At the time of the slaughter, blood samples were collected into empty tubes (Vacutainer, BD, USA) via jugular vein puncture. All analyses of the blood, except retinol and tocopherol, were performed at 37 °C with a clinical auto-analyser (ILAB-650; Instrumentation Laboratory, Werfen, Milan, Italy). The concentrations of albumins, globulins, total protein, cholesterol, total bilirubin, *γ*-glutamyl transferase (GGT), and glutamate oxaloacetate transaminase (GOT) were determined using commercial kits from Instrumentation Laboratory. Haptoglobin and ceruloplasmin were analysed using the methods described by Skinner et al. (39) and Sunderman and Nomoto (40), respectively, adapted to the ILAB-650. Paraoxonase (PON) activity was assessed based on an adaptation of the method originally described by Ferré et al. (41), as described by Bionaz et al. (42). Ferric reducing antioxidant power (FRAP) was analysed by adapting a colorimetric method proposed by Benzie and Strain (43) to the ILAB-650. Plasma thiol groups were determined using the Plasma Thiol Group Test (Diacron, Grosseto, Italy) adapted to the ILAB-650. Plasma retinol and tocopherol were extracted with hexane and analysed by reverse-phase HPLC using an Allsphere ODS-2 column (3 μm, 150 × 4.6 mm; Grace Davison Discovery Sciences, Deerfield, IL) and a 80:20 (v/v) mixture of methanol and tetrahydrofuran as the mobile phase. The compounds were detected with an ultraviolet (UV) detector set at 325 nm (for vitamin A), 290 nm (for vitamin E), or 460 nm (for β-carotene) (44).

Liver and sternocleidomastoid muscle tissue samples were collected to evaluate the antioxidant status. The samples were lyophilised using a freeze-dryer, ground with a homogeniser, and stored at −20 °C until use. Then, 0.5 g of minced sample was mixed with 25 mL of a methanol/water 80/20 (v/v) solution, sonicated at 60 °C for 30 min, and centrifugated at 6000 rpm for 5 min. The supernatants were used for the antioxidant analyses.

The 2,2-diphenyl-1-picrylhydrazyl (DPPH) radical scavenging activity was determined according to Nenadis et al. (32) and a UV-1800 spectrophotometer. The per cent radical scavenging activity (%RSA) was determined using the following formula:


$$\%\mathrm{RSA}=[\mathrm{Ab}s_{515(t=0)}–\mathrm{Ab}s_{515(t)}]/[\mathrm{Ab}s_{515(t=0)}]\times 100$$

After correction with the appropriate blank. This value was converted to Trolox equivalents with a calibration curve (y = 0.6439x + 1.6609, R^2^ = 0.996). The results are presented as the mean ± standard deviation of %RSA.

The 2,2′-azino-bis(3-ethylbenzothiazoline-6-sulfonic acid) (ABTS) radical scavenging activity was evaluated according to the protocol reported by Re et al. (45) and adjusted according to Nenadis et al. (32). The per cent inshibition of the ABTS radical cation (% Inh) was calculated with the formula:


$$\%\mathrm{Inh}=[\mathrm{Ab}s_{734(t=0)}–\mathrm{Ab}s_{734(t)}]/[\mathrm{Ab}s_{734(t=0)}]\times 100$$

After correction with the appropriate blank. This value was converted to Trolox equivalents with a calibration curve (y = 2.8169x – 0.1910, *R*^2^ = 0.999). The results are presented as the mean ± standard deviation of μmol Trolox/g dry sample.

The cupric ion reducing antioxidant (CUPRAC) capacity was measured according to the protocol described by Apak et al. (46). The absorbance at 450 nm was recorded after incubating the solution in the dark for 30 min. The data were converted to Trolox equivalents with a calibration curve (y = 0.0041x – 0.1101, R^2^ = 0.997). The results are presented as the mean ± standard deviation of μmol Trolox/g dry sample. In all the assays, measurements were performed in triplicate.

### 2.7 Meat proximate analysis

The meat analyses were performed on six pigs (two from each replicate) randomly chosen for each group. The protein, lipid, moisture, and ash contents in the sternocleidomastoid muscle were determined according to AOAC methods 992.15, 960.30, 950.46, and 923.03, respectively.

### 2.8 Meat quality evaluation

#### 2.8.1 Raw meat analysis

The gluteus medius muscle (from the hindleg) was chosen for analysis because it is usually used to produce dry-cured ham. Twenty-four hours after slaughtering, the pH was measured using a penetrating electrode connected to a portable pH-meter (Mod pH25, Crison, Barcelona, Spain). Colour measurements were conducted after blooming for 1 h at 4°C ± 1 °C (47) using a Minolta Chromameter CR400 with the D65 light source (Minolta, Osaka, Japan). The lightness (L*), redness (a*), and yellowness (b*) indexes were determined.

#### 2.8.2 Cooked meat analysis

Cooking loss of the gluteus medius muscle was assessed based on the study by Honikel (47). Samples (6.0 × 6.0 × 2.5 cm; average weight: 83.32 ± 1.67 g for the C group, 84.46 ± 4.15 g for the P-LOW group, and 84.05 ± 2.92 g for the P-HIGH group) were cooked in plastic bags in a water bath (80 °C for 1 h). Then, the samples were cooled under running tap water for 30 min. Cooking loss was calculated as:


100×(initial weight–final weight)/initial weight

Shear force was measured by following the Warner–Bratzler shear force protocol described by Honikel (47). Three cylindrical cores (with a diameter of 1.25 cm) were obtained from the muscle samples used to assess cooking losses (cut parallel to the gluteal muscle fibres). The protocol used a Warner–Bratzler shear device fitted to a texture analyser (TVT6700, Perten Instrument, Segeltorp, Sweden). The peak force is expressed as kg/cm^2^.

#### 2.8.3 Subcutaneous fat

The pH and colour of the subcutaneous fat covering the gluteus medius was measured at the posterior part of the back (the same anatomical location from which the meat samples were obtained). The pH (at 24 h after slaughter) and colour measurements (L*, a*, and b*) were carried out as described in section 2.8.1. To avoid oxidation, measurements were conducted before the blooming period.

### 2.9 Statistical analysis

The performance parameters were analysed with a simple hierarchical analysis of variance (ANOVA) model, with the replicates (pen) nested within the diet and initial body weight used as a covariate. In particular, the growth performance data were analysed with the following linear model:


$$y_{\mathrm{ik}}=\mu +D_{i}+I_{k}(D)+e_{\mathrm{ikj}}$$

where y_ik_ is the observation, *μ* is the overall mean, D_i_ is the fixed effect of the i^th^ diet (i = 1–3), I_k_ is the random effect of the replicate nested within the treatment (*k* = 1–3) and e_ikj_ is the residual error.

Backfat thickness was analysed with ANOVA, while the chi-square test was used to compare the pubertal rate among the groups. Fischer’s exact test was used to analyse the differences in the binomial (yes/no) morphological and pathological factors among the groups. The Kruskal–Wallis test was employed to evaluate differences in various histological parameters among the groups. The antioxidant data were first analysed with the Shapiro–Wilk test to assess normality. Normally distributed data were analysed with one-way ANOVA, followed by the *t*-test for pairwise comparisons. Non-normally distributed data were analysed with the Kruskal–Wallis test, followed by the Wilcoxon test for pairwise comparisons.

The blood serum parameters, quality traits, texture properties and proximate analysis were analysed with one-way ANOVA, with D as fixed factor, followed by Tukey’s test:


$$y_{\mathrm{ik}}=\mu +D_{i}+e_{\mathrm{ik}}.$$

Several blood serum parameters (cholesterol, GOT, and thiol groups) were log-transformed to normalise their distribution and stabilise the measurements variance. A multivariate analysis of variance (MANOVA) was then performed with the feeding group as the fixed factor and Wilks’ *λ* as the test statistic. For these data, a canonical linear discriminant analysis (LDA) was applied to derive canonical discriminant functions that maximized between-group separation. The performance of the discriminant rule was evaluated by leave-one-out cross-validation, calculating the proportion of correctly classified animals. To assess whether the observed accuracy exceeded that expected by chance, a permutation test was conducted. Group labels were randomly shuffled (e.g., 999 permutations), refitting the LDA each time and recomputing the cross-validated accuracy. The permutation *p*-value was defined as the proportion of permuted accuracies greater than or equal to the observed accuracy. The R software was employed for analyses (49).

## 3 Results

### 3.1 Pig performance

The BW, ADG, FCR, HCW, and dressing out percentage did not differ between the experimental groups (Table 2). Feed intake was the same for all groups; the pigs always ingested the entire amount of feed provided throughout the trial.

**Table 2:** Growth performance parameters and carcass characteristics observed in the control group and the OMWW polyphenol-supplemented groups.

Items	Groups^5^			*p*-value
	Control	P-LOW	P-HIGH	
Initial BW^1^ (kg)	98.48 ± 3.5	103.5 ± 6.3	109.09 ± 4.3	0.47
Final BW^1^ (kg)	145.7 ± 16.7	145.2 ± 11.7	153.4 ± 13.6	0.07
ADG^2^ (g)	673.97 ± 0.3	536.52 ± 0.1	639.13 ± 0.2	0.15
FCR^3^	4.2 ± 0.3	5.2 ± 0.1	4.4 ± 0.2	0.52
HCW^4^ (kg)	120.6 ± 13.7	119.5 ± 9.5	126.3 ± 12.3	0.39
Carcass yield (%)	82.95 ± 0.1	80.79 ± 0.3	83.79 ± 0.2	0.09

1 BW, body weight.

2 ADG, average daily gain.

3 FRC, feed conversion ratio.

4 HCW, hot carcass weight.

5 C, control group fed basal diet; P-L, control group fed basal diet supplemented with 74 ppm OMWW polyphenols; P-HIGH, control group fed basal diet supplemented with 225 ppm OMWW polyphenols.

### 3.2 Fat deposition

Fat deposition did not differ among the groups (*p* = 0.35; Table 3).

**Table 3:** Backfat thickness (cm) observed at the slaughtering.

Groups^1^	Mean	SD^2^	Min	Max
C	2.38	0.90	0.82	3.75
P-LOW	2.36	0.60	1.37	3.19
P-HIGH	1.91	0.64	0.85	2.97

1 C, control group fed basal diet; P-HIGH, control group fed basal diet supplemented with 74 ppm OMWW polyphenols; P-HIGH, control group fed basal diet supplemented with 225 ppm OMWW polyphenols.

2 SD, standard deviation.

### 3.3 Histopathology and morphometry

There were no differences in the parameters assessed for the liver, muscle, fat, and reproductive tract tissue samples among the groups. A tissue representative figure is available as supplementary material (Supplementary Figure 1) to document tissue morphology without overloading the main manuscript. The histology scores are available as Supplementary material (Supplementary Table 1).

### 3.4 Pubertal status

At slaughter, pre-pubertal and pubertal gilts presented clearly distinguishable reproductive tracts. Pre-pubertal animals exhibited small, smooth ovaries, and short (<90 cm) uterine horns, whereas pubertal gilts showed longer (>150 cm) uterine horns. In pubertal animals, the ovarian surface was fully occupied by follicles >6 mm in diameter and corpora lutea. There were no significant differences in the percentage of animals that reached puberty by the end of the observation period between the groups (C vs. P-LOW, *p* = 0.61; CON vs. P-HIGH, *p* = 0.90; Table 4).

**Table 4:** Numbers of pubertal and prepubertal gilts at the final stage in each treatment group, as identified at inspection after slaughter and isolation of the reproductive tracts.

	Groups^1^		
	C	P-LOW	P-HIGH
Pubertal gilts, *n*^2^ (%)	5 (29.4)	3 (21.4)	3 (27.3)
Prepubertal gilts, *n*	12	11	8

1 C, control group fed basal diet; P-LOW, control group fed basal diet supplemented with 74 ppm OMWW polyphenols; P-HIGH, control group fed basal diet supplemented with 225 ppm OMWW polyphenols.

2 *n*, number of animals.

### 3.5 Blood serum parameters

**Figure 1:**
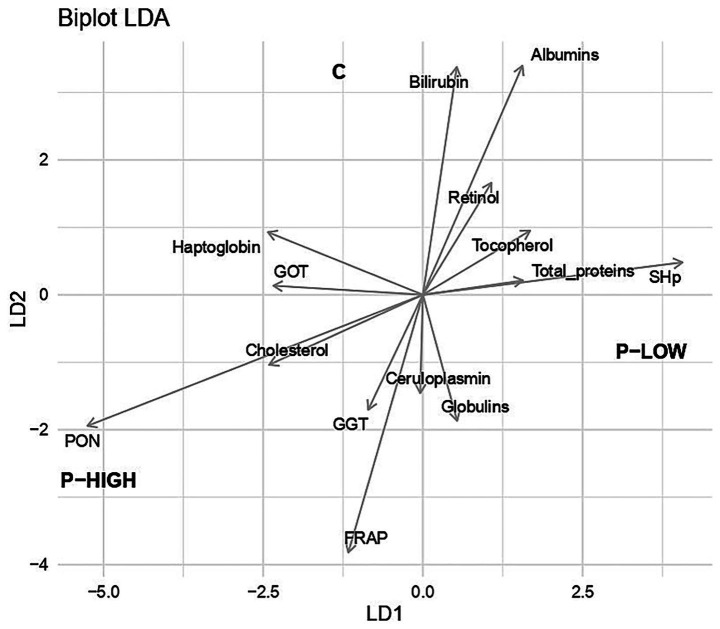
Linear discriminant analysis (LDA) diagram representing differentiations of blood serum parameters by the three experimental groups: C (control), P-LOW (C diet supplemented with 74 ppm OMWW polyphenols), and P-HIGH (C diet supplemented with 225 ppm OMWW polyphenols). SHp, Thiol groups; PON, paraoxonase; FRAP, ferric reducing ability of plasma; GOT, aspartate aminotransferase; GGT, gamma-glutamyl transferase.

Among the evaluated blood serum parameters, there was a tendency for higher PON activity and GOT levels in the P-HIGH group compared with the P-LOW group (*p* = 0.06; Table 5). There were no differences in the other parameters among the groups. Multivariate analysis of variance showed an overall effect of the diet on the biochemical profile [Wilks’ *λ* = 0.27, approx. F (28,54) = 1.80, *p* = 0.032]. Figure 1 shows the outcome of the canonical discriminant analysis. The first discriminant function was statistically significant [canonical correlation = 0.77, Wilks’ λ = 0.27, χ^2^(28) = 44.1, *p* = 0.027], indicating a measurable, though modest, separation among the three experimental groups. Under leave-one-out cross-validation, the LDA correctly classified 51.2% of animals, compared with 41.9% expected by always predicting the most frequent group. A permutation test (999 permutations) indicated a trend toward above-chance classification performance (mean permuted accuracy 35.7% ± 8.6%, permutation *p* = 0.058), suggesting that although some discriminative structure is present, the overall ability of the model to reliably distinguish the groups remains limited.

**Table 5:** Blood serum parameters of pigs from control and polyphenol-supplemented groups.

Parameter	Groups^1^			*p*-value
	C	P-LOW	P-HIGH	
Albumins (g/L)	38.1 ± 0.8	35.8 ± 0.8	38.4 ± 1.1	0.07
Total proteins (g/L)	76.2 ± 1.2	77.7 ± 1.2	78.0 ± 1.6	0.53
Globulins (g/L)	37.9 ± 1.3	41.4 ± 1.3	39.0 ± 1.7	0.11
Cholesterol (mmol/L)	2.7 ± 0.1	2.8 ± 0.1	2.7 ± 0.1	0.94
GOT^2^ (U/L)	61.1 ± 5.1	49.3 ± 5.1	67.3 ± 7.5	0.06
GGT^3^ (U/L)	38.5 ± 2.2	37.9 ± 2.1	38.3 ± 2.9	0.97
Bilirubin (mcmol/L)	1.8 ± 0.7	2.4 ± 0.6	1.2 ± 0.7	0.23
Haptoglobin (g/L)	0.8 ± 0.04	0.8 ± 0.04	0.9 ± 0.06	0.70
Ceruloplasmin(mcmol/L)	16.7 ± 0.7	16.8 ± 0.7	18.2 ± 0.9	0.33
FRAP^4^ (mcmol/L)	159 ± 6.0	162 ± 6.0	158 ± 8.0	0.85
Thiol groups (mcmol/L)	253.2 ± 14.3	248.6 ± 14.2	223.6 ± 19.0	0.49
Paraoxonase (PON^5^, U/L)	51.7 ± 2.6	49.1 ± 2.6	58.9 ± 3.5	0.06
Retinol (mcg/100 mL)	25.2 ± 1.9	23.7 ± 1.9	25.5 ± 2.5	0.77
Tocopherol (mg/100 mL)	3.1 ± 0.1	3.4 ± 0.1	3.1 ± 0.1	0.11

1 C, control group fed basal diet; P-LOW, control group fed basal diet supplemented with 74 ppm OMWW polyphenols; P-HIGH, control group fed basal diet supplemented with 225 ppm OMWW polyphenols.

2 GOT, Aspartate aminotransferase.

3 GGT, Gamma-glutamyl transferase.

4 FRAP, Ferric reducing ability of plasma.

5 PON, Paraoxonase.

### 3.6 Antioxidant activity in muscle and liver

**Figure 2:**
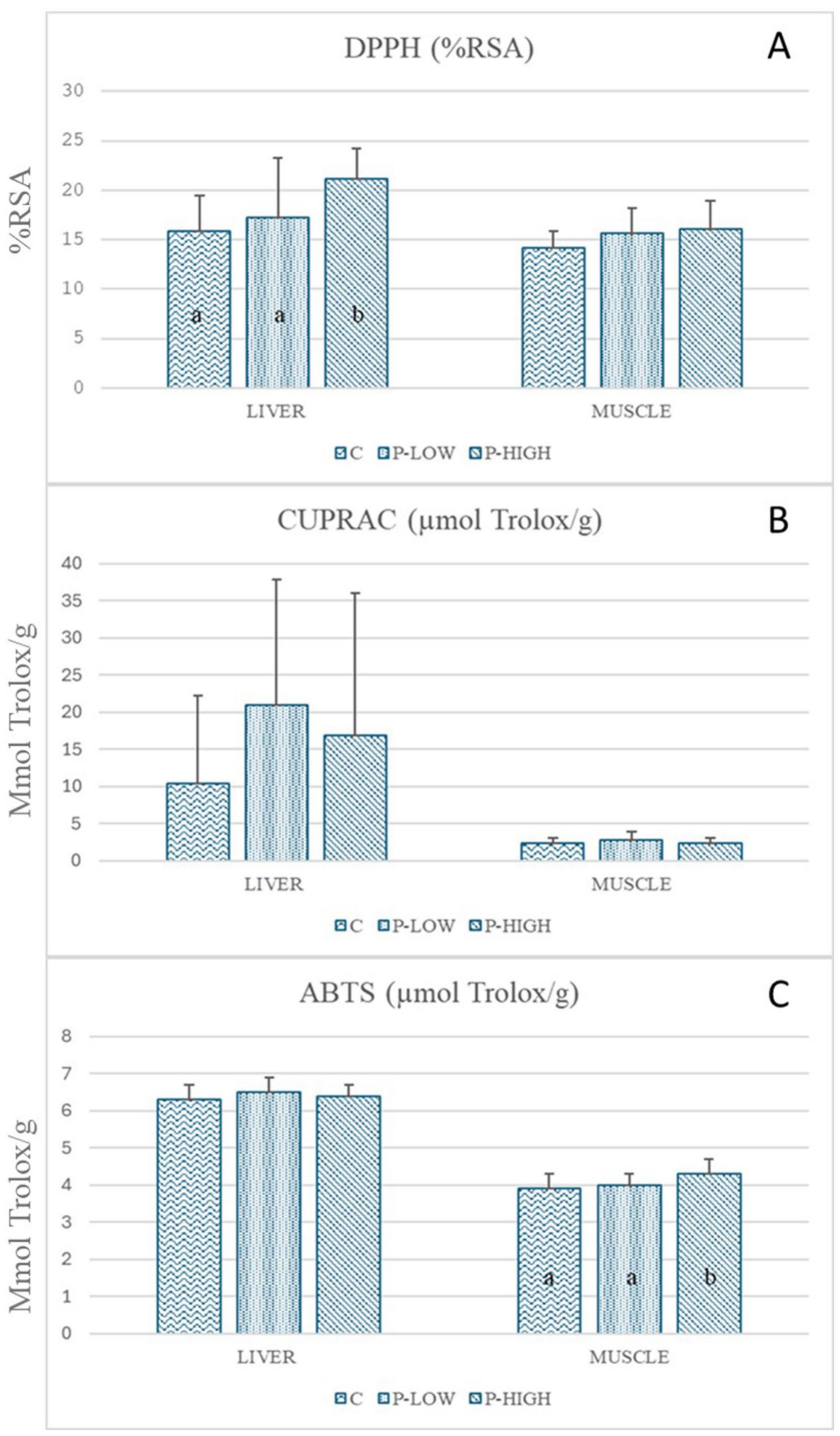
DPPH, CUPRAC, and ABTS assays in liver and muscle among the three experimental groups: C (control), P-LOW (C diet supplemented with 74 ppm OMWW polyphenols), and P-HIGH (C diet supplemented with 225 ppm OMWW polyphenols); (a, b) *p* ≤ 0.05.

In the liver, the DPPH radical scavenging assay showed a significant (*p* = 0.019) difference in % RSA among the groups, with an increase in the P-HIGH group compared with the C and P-LOW groups (Figure 2A). There were no differences (*p* > 0.05) among the groups in the muscle. There were no differences in the CUPRAC assay among the groups in the muscle and liver (Figure 2B). Finally, ABTS radical scavenging activity did not differ among the groups in the liver, but in the muscle, it was higher in the P-HIGH group compared with the C and P-LOW groups (Figure 2C). All assays for liver and muscle were reported in Figure 2.

### 3.7 Meat proximate composition

The proximate composition (protein, lipid, moisture, and ash contents) of the meat did not differ among the groups (Table 6).

**Table 6:** Proximate composition analyses of meat (*sternocleidomastoid*) from pigs fed control or polyphenol-supplemented diets.

Items	Groups^1^			SEM^2^	*p*-value
	C	P-LOW	P-HIGH		
Protein (%)	21.10	21.27	20.94	0.163	0.392
Lipid (%)	1.14	1.13	1.16	0.019	0.648
Moisture (%)	70.52	70.26	70.37	0.202	0.687
Ashes (%)	7.23	7.32	7.52	0.094	0.121

1 C, control group fed basal diet; P-LOW, control group fed basal diet supplemented with 74 ppm OMWW polyphenols; P-HIGH, control group fed basal diet supplemented with 225 ppm OMWW polyphenols.

2 SEM, standard error of the mean.

### 3.8 Meat and subcutaneous fat quality

Table 7 presents the meat and subcutaneous fat quality results. There were no differences among the groups regarding the pH and L* values of the meat. The a* index was decreased in the P-LOW and P-HIGH groups (with the lowest value in the P-LOW group and the highest value in the C group). The b* index was higher in the P-HIGH group compared with the C group.

**Table 7:** Quality traits of meat and subcutaneous fat of pigs fed control or polyphenol-supplemented diets.

Items	Groups^1^			SEM^2^	p-value
	C	P-LOW	P-HIGH		
*Gluteus medius*					
pH (24 h)	5.47	5.47	5.45	0.031	0.919
L*^3^	50.76	50.96	52.36	0.765	0.282
a*^4^	9.24^a^	7.59^b^	8.99^ab^	0.426	0.018
b*^5^	4.59^a^	5.36^ab^	5.77^b^	0.269	0.011
Cooking loss (%)	34.50^a^	32.20^b^	33.72^ab^	0.535	0.025
WB shear force (kg/cm^2^)^6^	62.90	69.11	60.97	3.956	0.323
*Subcutaneous fat*					
pH (24 h)	5.83	5.80	5.90	0.030	0.067
L*^3^	71.37^ab^	73.86^a^	70.67^b^	0.756	0.015
a*^4^	6.07^a^	4.37^b^	5.19^ab^	0.370	0.011
b*^5^	6.56	5.74	6.03	0.285	0.137

1 C, control group fed basal diet; P-LOW, control group fed basal diet supplemented with 74 ppm OMWW polyphenols; P-HIGH, control group fed basal diet supplemented with 225 ppm OMWW polyphenols.

2 SEM, Standard Error of the Mean.

3 L*, lightness.

4 a*, redness.

5 Yellowness.

6 Warner Bratzler shear force.

Cooking loss was decreased in the P-LOW and P-HIGH groups compared with the C group. However, Warner–Bratzler shear force did not differ between the groups.

For the subcutaneous fat, pH did not differ significantly between the groups. However, there were differences in the colour parameters: L* was increased and a* was decreased in the P-LOW group compared with the C group. On the other hand, b* did not differ between the groups.

## 4 Discussion

OMWW and its phenolic extracts have recently been tested *in vitro* (12, 48, 49, 50) and *in vivo* (5, 16, 28) for their potential application as supplements for feed used for livestock, including pigs and poultry. The importance of phenolic compounds is mainly related to their antioxidant role (20). Furthermore, olive polyphenols are highly water soluble and thus mainly dispersed in the wastewater (51). In our study, we tested two doses of an OMWW phenolic extract as feed supplements in heavy female finishing pigs, and evaluated the impact on growth performance, morphological characteristics, the oxidative status, and meat quality.

### 4.1 Animal performance and carcass traits

Growth performance parameters did not significantly differ between the three experimental groups (Table 2). Although not reaching significance, pigs of P-HIGH group had the highest final BW (+5.3% compared to C). Several studies reported that olive cake, one of the most used olive by-product in animal nutrition, failed to influence growth performance when added to pigs’ diet (22, 27, 52, 53). Similarly, no differences were found in the BW of sows after a dietary inclusion of olive pulp (17) as well as in pigs supplemented with an oleuropein extract (24) and partially defatted olive cake (52). However, a recent study showed that a dietary treatment with OMWW increased BW and ADG in piglets (28). Similarly, Liotta et al. (23) reported that final BW and ADG of pigs increased when they were supplemented with olive cake. Thus, our results are in line with the literature showing no strong evidence of improved performance.

Regarding backfat thickness measured at slaughtering literature is not consistent. In our study, no differences were found between experimental groups, as reported also in two recent studies with olive pulp dietary inclusion in gestating sows (17) or olive cake supplementation in Bísaro pigs (54). Nevertheless, Palma-Granados et al. (55) reported a lesser fat deposition after dietary pigs’ supplementation with dry olive pulp, compared to wet crude olive cake and control diets.

### 4.2 Histopathological and morphometric findings

The absence of macroscopic and microscopic tissue changes in all examined tissues (liver, ovary, uterus, adipose tissue, and skeletal muscle) from all groups suggests that OMWW polyphenol supplementation has no or a very limited effect on tissue morphology. There were no differences in the onset of puberty among the groups (Table 4). Nevertheless, there have been reports that other plant polyphenols have a specific chemical structure that could modulate the synthesis of follicle-stimulating hormones, steroid hormones (e.g., progesterone), and prostaglandins, which are involved in follicular growth and development of the ovary (55, 56). Given the lack of effects in this study, the efficacy of polyphenols may differ depending on their type.

### 4.3 Oxidative status in the blood

The oxidative status in the blood serum was evaluated considering several parameters. A tendency (*p* = 0.06) was found for an increase in PON in the P-HIGH group compared with the others (Table 5). The paraoxonase-1 (PON1) is synthesized in the liver and then secreted into the bloodstream. Its biosynthesis and activity are influenced by the oxidative conditions (57) and reduced PON1 levels are associated with hepatic inflammation and oxidative stress. In the presence of natural antioxidants, such as polyphenols, serum PON1 activity generally increases (57, 58). A recent study showed that PON1 activity was higher in sows that received supplementation with a mixture containing natural polyphenols (59) with respect to the control condition. Moreover, Israr et al. (60) reported that PON1 was increased in broiler chickens after dietary inclusion of grape seed powder (a source of polyphenols) and zinc. Indeed, the multivariate analysis indicates that the experimental treatments influenced the overall biochemical profile, even if the magnitude of this effect was only moderate. Consistent with this, inspection of the discriminant loadings indicated that PON, FRAP and ceruloplasmin contributed to the separation of the treated groups from control (Figure 1). The first canonical variate primarily distinguished the P-HIGH group from all others, with this separation mostly driven by differences in PON concentration. From a predictive standpoint, the performance of the LDA was relatively modest and the permutation test yielded only a borderline *p*-value (*p* = 0.058). Therefore, the LDA results should be regarded primarily as descriptive and exploratory, serving to visualize multivariate patterns and highlight variables contributing to group differentiation, rather than as evidence of a robust diagnostic tool for individual-level classification.

### 4.4 Antioxidant activity in liver and muscle

We evaluated the antioxidant activity of the sternocleidomastoid muscle based on a series of assays (DPPH, CUPRAC, and ABTS). For the DPPH assay, which measures antioxidant activity by exploiting the electron/hydrogen transfer ability of molecules, there was higher radical scavenging activity in the liver of the P-HIGH group compared with the C group (Figure 2A). There was also a difference in DPPH radical scavenging between the livers of the P-HIGH and P-LOW groups, suggesting that a higher dose of the OMWW phenolic extract could exert better antioxidant effects. To the authors’ knowledge, there have been no studies on antioxidant activity in pigs fed olive polyphenols. Vasilopoulou et al. (61) examined DPPH radical scavenging activity in the liver and muscle of broilers following supplementation with an olive leaf extract, but there were no differences between the groups. The authors justified these results based on the nature of the extract, which contained a large amount of oleuropein, known to be a pro-oxidant when given at high doses (62). King et al. (63) also reported no significant changes in the antioxidant activity of poultry meat after dietary supplementation with an olive freeze-dried powder containing 2.5% hydroxytyrosol. In contrast, Branciari et al. (20) reported increased antioxidant activity (based on the DPPH assay) in poultry meat after dietary supplementation with semi-solid olive cake. Moreover, Jang et al. (64) noted higher scavenging activity in breast meat from broilers fed an herbal extract mix of antioxidants compared with the control. These inconsistencies in DPPH radical scavenging could be related to several factors, such as the nature of the olive by-products used, the storage conditions, their polyphenol profile and concentrations (which may vary depending on environment and cultivar), and the level of dietary inclusion.

The CUPRAC assay, which is based on the reduction of copper, showed no differences in the muscle and liver among the groups (Figure 2B), and the ABTS assay showed no difference in the liver among the groups (Figure 2C). Consistently, Untea et al. (65) did not find increased antioxidant activity in the liver of pigs subjected to dietary supplementation with a plant mixture rich in polyphenols. However, ABTS radical scavenging activity was modestly but significantly higher in the muscle of the P-HIGH group compared with the muscle of the C group (Figure 2C). A possible explanation for this finding could be related to the muscle matrix itself. To our knowledge, there have been no studies that used the ABTS assay to assess the antioxidant status of the muscle of pigs or other monogastric species.

In summary, the variations in the results of the three antioxidant assays could be related to the antioxidant capacity of each polyphenol (66). Moreover, there has been very limited research on how olive polyphenols alter the antioxidant capacity of tissues, especially the liver, and the available results have often been inconsistent. Therefore, additional detailed studies are required to investigate the positive effects of polyphenols on the tissues and metabolism of monogastric animals.

### 4.5 Meat and subcutaneous fat quality

Among meat quality traits, the measured pH of the gluteus medius was in the expected range for pork (5.5–6.0) (67), and there were no differences among the groups (Table 7). The decrease in a* in the raw meat from the P-LOW and P-HIGH groups (Table 7) is consistent with a study that involved feeding pigs partially defatted olive cake (52). In addition, other studies have reported that grape and olive oil extracts increased the redness of meat during preservation (68, 69). Dietary antioxidants are known for their ability to protect myoglobin, whose oxidation and reduction are related to meat discolouration (70, 71). Oxymyoglobin, responsible for the red colour of meat, is unstable and can be easily oxidised to metmyoglobin, becoming brownish in colour (72). Furthermore, the oxidation of haem-containing proteins promotes the formation of superoxide anions, which have detrimental effects on meat quality (72, 73). Polyphenols could potentially protect oxymyoglobin from oxidation, but their ability to interact with haem-containing proteins depends on their chemical structure, the dose, the pH, and/or possible interferences with transition metal ions (73). For example, certain polyphenols, including hydroxytyrosol (and not all polyphenols) have demonstrated a pro-oxidant action on oxymyoglobin *in vitro* (72). We also noted higher b* values as the OMWW polyphenol dose increased; however, this variation could also be related to the natural brownish colour of the extract. Indeed, pigments in olives, such as melanin and humic acid-like substances, can be transferred to OMWW (which commonly has a brown colour) and therefore can be found in the extracts (74). Consistently, Moroney et al. (75) reported that pork patties containing a seaweed extract that is brown in colour appeared more brown than fresh pork patties.

Cooking loss is related to the water retention capacity of the muscle and, consequently, to meat quality characteristics (i.e., tenderness) (76, 77). A decrease in cooking loss means a higher water-holding capacity and, consequently, better meat quality and a longer shelf life (78). Cooking loss decreased in the P-HIGH and P-LOW groups compared with the C group (although the difference was significant only for the P-LOW group). Xu et al. (79) examined pork meatballs and reported that certain water-soluble polyphenols (i.e., gallic acid, epigallocatechin gallate, and tannic acid) enhance the stability of myofibrillar proteins in gels. Similarly, hydroxytyrosol and tyrosol, the main polyphenols contained in the OMWW extract, can interact with water and other polar compounds through hydrogen bonding and potentially reduce the hydrophobic surface of myofibrils, maintaining their strength and thus reducing cooking loss. We noted that the L* values of the meat were not affected the supplementation with the OMWW extract, consistent with other reports (23, 31, 52). Although dietary antioxidants are not thought to influence this parameter, Joven et al. (22) reported a decrease in L* in response to increasing levels of olive cake in the diet of finishing pigs. Furthermore, meat becomes dark when it retains more water (80). Additional investigation on the colour parameters of subcutaneous fat is recommended.

## 5 Conclusion

Supplementing the diet of female finishing pigs with two different doses of OMWW polyphenols (74 and 225 ppm) did not negatively affect productive performance. These phenols (particularly the higher dose) enhanced the liver and muscle antioxidant status, confirmed by a tendency for increased PON activity in the blood. Nevertheless, it is hard to interpret the findings given the scarcity of data that has been published. The OMWW-derived polyphenols also enhanced meat quality by increasing the water retention capacity. In summary, our findings seem to confirm dose-dependent effects of OMWW polyphenols and variability depending on the kind of extract or polyphenols. It is advisable that more studies be undertaken to address these issues.

## Data Availability

The raw data supporting the conclusions of this article will be made available by the authors, without undue reservation.
