# Supplementary material for: Effects of dietary supplementation with an olive mill wastewater phenolic extract on the growth performance, oxidative status, and meat quality traits of finishing pigs
Source: Front Vet Sci. 2026 Feb 24;13:1761378. doi: 10.3389/fvets.2026.1761378 (PMC12974233; doi:10.3389/fvets.2026.1761378)
Supplement: Supplementary file 1 [file Table_1.docx]

**Supplementary Table 1. Summary of the data related to the histologic evaluation of skeletal muscle and fat tissue and macroscopic and histologic evaluation of the reproductive system (ovary and uterus).**

| **Case** | **Liver** | | | | **Skeletal muscle** | | | | | | **Fat tissue** | | | **Repr.** | **Ovary** | | | **Uterus** | | | | | |
| --- | --- | --- | --- | --- | --- | --- | --- | --- | --- | --- | --- | --- | --- | --- | --- | --- | --- | --- | --- | --- | --- | --- | --- |
|  | **Deg.** | **Necr.** | **Leuk.** | **Fibr.** | **Deg.** | **Necr.** | **Atrop.** | **Hypert.** | **Regen.** | **Leuk.** | **Necr.** | **Atrop.** | **Leuk.** | **Status*** | **FL** | **LB** | **Leuk.** | **Deg.** | **Necr.** | **ADM** | **Leuk.** | **Fibr.** |  |
| **Control** | 0 | 0 | 0 | 0 | 0 | 0 | 0 | 0 | 0 | 0 | 0 | 0 | 0 | P | Y | Y | 0 | 0 | 0 | N | 0 | 0 |  |
|  | 0 | 0 | 1 | 0 | 0 | 0 | 0 | 0 | 0 | 0 | 0 | 0 | 0 | NP | Y | N | 0 | 0 | 0 | N | 0 | 0 |  |
|  | 0 | 0 | 0 | 0 | 0 | 0 | 0 | 0 | 0 | 0 | 0 | 0 | 0 | NP | Y | N | 0 | 0 | 0 | N | 0 | 0 |  |
|  | 0 | 0 | 0 | 0 | 0 | 0 | 0 | 0 | 0 | 0 | 0 | 0 | 0 | NP | Y | N | 0 | 0 | 0 | N | 0 | 0 |  |
|  | 0 | 0 | 1 | 0 | 0 | 0 | 0 | 0 | 0 | 0 | 0 | 0 | 0 | P | Y | Y | 0 | 0 | 0 | N | 0 | 0 |  |
|  | 0 | 0 | 0 | 0 | 0 | 0 | 0 | 0 | 0 | 0 | 0 | 0 | 0 | P | Y | Y | 0 | 0 | 0 | N | 0 | 0 |  |
|  | 0 | 0 | 1 | 0 | 0 | 0 | 0 | 0 | 0 | 0 | 0 | 0 | 0 | NP | Y | N | 0 | 0 | 0 | N | 0 | 0 |  |
|  | 0 | 0 | 2 | 0 | 0 | 0 | 0 | 0 | 1 | 0 | 0 | 0 | 0 | NP | Y | N | 0 | 0 | 0 | N | 0 | 0 |  |
|  | 1 | 0 | 0 | 0 | 0 | 0 | 0 | 0 | 0 | 0 | 0 | 0 | 0 | NP | Y | N | 0 | 0 | 0 | N | 0 | 0 |  |
|  | 0 | 0 | 0 | 0 | 0 | 0 | 0 | 0 | 0 | 0 | 0 | 0 | 0 | NP | Y | N | 0 | 0 | 0 | N | 0 | 0 |  |
|  | 0 | 0 | 0 | 0 | 0 | 0 | 0 | 0 | 0 | 0 | 0 | 0 | 0 | NP | Y | N | 0 | 0 | 0 | N | 0 | 0 |  |
|  | 0 | 0 | 1 | 0 | 0 | 0 | 0 | 0 | 1 | 0 | 0 | 0 | 0 | P | Y | Y | 0 | 0 | 0 | N | 0 | 0 |  |
|  | 1 | 0 | 0 | 0 | 0 | 0 | 0 | 0 | 0 | 0 | 0 | 0 | 0 | NP | Y | N | 0 | 0 | 0 | N | 0 | 0 |  |
|  | 0 | 0 | 0 | 0 | 0 | 0 | 0 | 0 | 0 | 0 | 0 | 0 | 0 | NP | Y | N | 0 | 0 | 0 | N | 0 | 0 |  |
|  | 0 | 0 | 0 | 0 | 0 | 0 | 0 | 0 | 0 | 0 | 0 | 0 | 0 | P | Y | Y | 0 | 0 | 0 | N | 0 | 0 |  |
| **P-LOW** | 0 | 0 | 1 | 0 | 0 | 0 | 0 | 0 | 0 | 0 | 0 | 0 | 0 | NP | Y | N | 0 | 0 | 0 | N | 0 | 0 |  |
|  | 0 | 0 | 1 | 0 | 0 | 0 | 0 | 0 | 0 | 0 | 0 | 0 | 0 | NP | Y | N | 0 | 0 | 0 | N | 0 | 0 |  |
|  | 0 | 0 | 1 | 0 | 0 | 0 | 0 | 0 | 0 | 0 | 0 | 0 | 0 | NP | Y | N | 0 | 0 | 0 | N | 0 | 0 |  |
|  | 0 | 0 | 1 | 0 | 0 | 0 | 0 | 0 | 0 | 0 | 0 | 0 | 0 | P | Y | Y | 0 | 0 | 0 | N | 0 | 0 |  |
|  | 0 | 0 | 0 | 0 | 0 | 0 | 0 | 0 | 0 | 0 | 0 | 0 | 0 | P | Y | Y | 0 | 0 | 0 | N | 0 | 0 |  |
|  | 1 | 0 | 0 | 0 | 0 | 0 | 0 | 0 | 0 | 0 | 0 | 0 | 0 | P | Y | Y | 0 | 0 | 0 | N | 0 | 0 |  |
|  | 0 | 0 | 0 | 0 | 0 | 0 | 0 | 0 | 0 | 0 | 0 | 0 | 0 | NP | Y | N | 0 | 0 | 0 | N | 0 | 0 |  |
|  | 0 | 0 | 1 | 0 | 0 | 0 | 0 | 0 | 0 | 0 | 0 | 0 | 0 | NP | Y | N | 0 | 0 | 0 | N | 0 | 0 |  |
|  | 0 | 0 | 0 | 0 | 0 | 0 | 0 | 0 | 0 | 0 | 0 | 0 | 0 | NP | Y | N | 0 | 0 | 0 | N | 0 | 0 |  |
|  | 0 | 0 | 0 | 0 | 0 | 0 | 0 | 0 | 0 | 0 | 0 | 0 | 0 | NP | Y | N | 0 | 0 | 0 | N | 0 | 0 |  |
|  | 1 | 0 | 0 | 0 | 0 | 0 | 0 | 0 | 0 | 0 | 0 | 0 | 0 | NP | Y | N | 0 | 0 | 0 | N | 0 | 0 |  |
|  | 1 | 0 | 0 | 0 | 0 | 0 | 0 | 0 | 0 | 0 | 0 | 0 | 0 | NP | Y | N | 0 | 0 | 0 | N | 0 | 0 |  |
|  | 1 | 0 | 1 | 0 | 0 | 0 | 0 | 0 | 0 | 0 | 0 | 0 | 0 | NP | Y | N | 0 | 0 | 0 | N | 0 | 0 |  |
|  | 1 | 0 | 1 | 0 | 0 | 0 | 0 | 0 | 0 | 0 | 0 | 0 | 0 | NP | Y | N | 0 | 0 | 0 | N | 0 | 0 |  |
|  | 0 | 0 | 0 | 0 | 0 | 0 | 0 | 0 | 0 | 0 | 0 | 0 | 0 | n.a | n.a | n.a | n.a | n.a | n.a | n.a | n.a | n.a |  |
| **P-HIGH** | 0 | 0 | 0 | 0 | 0 | 0 | 0 | 0 | 0 | 0 | 0 | 0 | 0 | NP | Y | N | 0 | 0 | 0 | N | 0 | 0 |  |
|  | 0 | 0 | 0 | 0 | 0 | 0 | 0 | 0 | 0 | 0 | 0 | 0 | 0 | P | Y | Y | 0 | 0 | 0 | N | 0 | 0 |  |
|  | 0 | 0 | 0 | 0 | 0 | 0 | 0 | 0 | 0 | 0 | 0 | 0 | 0 | NP | Y | N | 0 | 0 | 0 | N | 0 | 0 |  |
|  | 1 | 0 | 0 | 0 | 0 | 0 | 0 | 0 | 0 | 0 | 0 | 0 | 0 | NP | Y | N | 0 | 0 | 0 | N | 0 | 0 |  |
|  | 0 | 0 | 1 | 0 | 0 | 0 | 0 | 0 | 0 | 0 | 0 | 0 | 0 | NP | Y | N | 0 | 0 | 0 | N | 0 | 0 |  |
|  | 1 | 0 | 1 | 0 | 0 | 0 | 0 | 0 | 0 | 0 | 0 | 0 | 0 | P | Y | Y | 0 | 0 | 0 | N | 0 | 0 |  |
|  | 0 | 0 | 1 | 0 | 0 | 0 | 0 | 0 | 0 | 0 | 0 | 0 | 0 | NP | Y | N | 0 | 0 | 0 | N | 0 | 0 |  |
|  | 0 | 0 | 0 | 0 | 0 | 0 | 0 | 0 | 0 | 0 | 0 | 0 | 0 | NP | Y | N | 0 | 0 | 0 | N | 0 | 0 |  |
|  | 0 | 0 | 0 | 0 | 0 | 0 | 0 | 0 | 0 | 0 | 0 | 0 | 0 | n.a | n.a | n.a | n.a | n.a | n.a | n.a | n.a | n.a |  |
|  | 0 | 0 | 0 | 0 | 0 | 0 | 0 | 0 | 0 | 0 | 0 | 0 | 0 | n.a | n.a | n.a | n.a | n.a | n.a | n.a | n.a | n.a |  |
|  | 0 | 0 | 0 | 0 | 0 | 0 | 0 | 0 | 0 | 0 | 0 | 0 | 0 | n.a. | n.a. | n.a. | n.a | n.a | n.a | n.a | n.a | n.a |  |
|  | 0 | 0 | 0 | 0 | 0 | 0 | 0 | 0 | 0 | 0 | 0 | 0 | 0 | n.a. | n.a. | n.a. | n.a | n.a | n.a | n.a | n.a | n.a |  |
|  | 0 | 0 | 0 | 0 | 0 | 0 | 0 | 0 | 0 | 0 | 0 | 0 | 0 | NP | Y | N | 0 | 0 | 0 | N | 0 | 0 |  |
|  | 0 | 0 | 0 | 0 | 0 | 0 | 0 | 0 | 0 | 0 | 0 | 0 | 0 | P | Y | Y | 0 | 0 | 0 | N | 0 | 0 |  |
|  | 0 | 0 | 0 | 0 | 0 | 0 | 0 | 0 | 0 | 0 | 0 | 0 | 0 | NP | Y | N | 0 | 0 | 0 | N | 0 | 0 |  |

Deg. = degeneration; Necr. = necrosis; Atrop. = atrophy; Hypert. = hypertrophy; Hyperp. = hyperplasia; Regen. = regeneration; Leuk. = leukocytic infiltrates; Fibr. = fibrosis; ADM = adenomyosis; FL = follicles; LT = luteal bodies; Y = present; N = Not present; Status* = reproductive status assessed macroscopically evaluating ovaries and uterus, where P = pubertal (presence of follicles larger than 6 mm and corpora lutea) and NP = non pubertal, n.a. = not available after dissection
