# Supplementary material for: Effects of dietary supplementation with an olive mill wastewater phenolic extract on the growth performance, oxidative status, and meat quality traits of finishing pigs
Source: Front Vet Sci. 2026 Feb 24;13:1761378. doi: 10.3389/fvets.2026.1761378 (PMC12974233; doi:10.3389/fvets.2026.1761378)
Supplement: Supplementary file 2 [file Image_1.pdf]

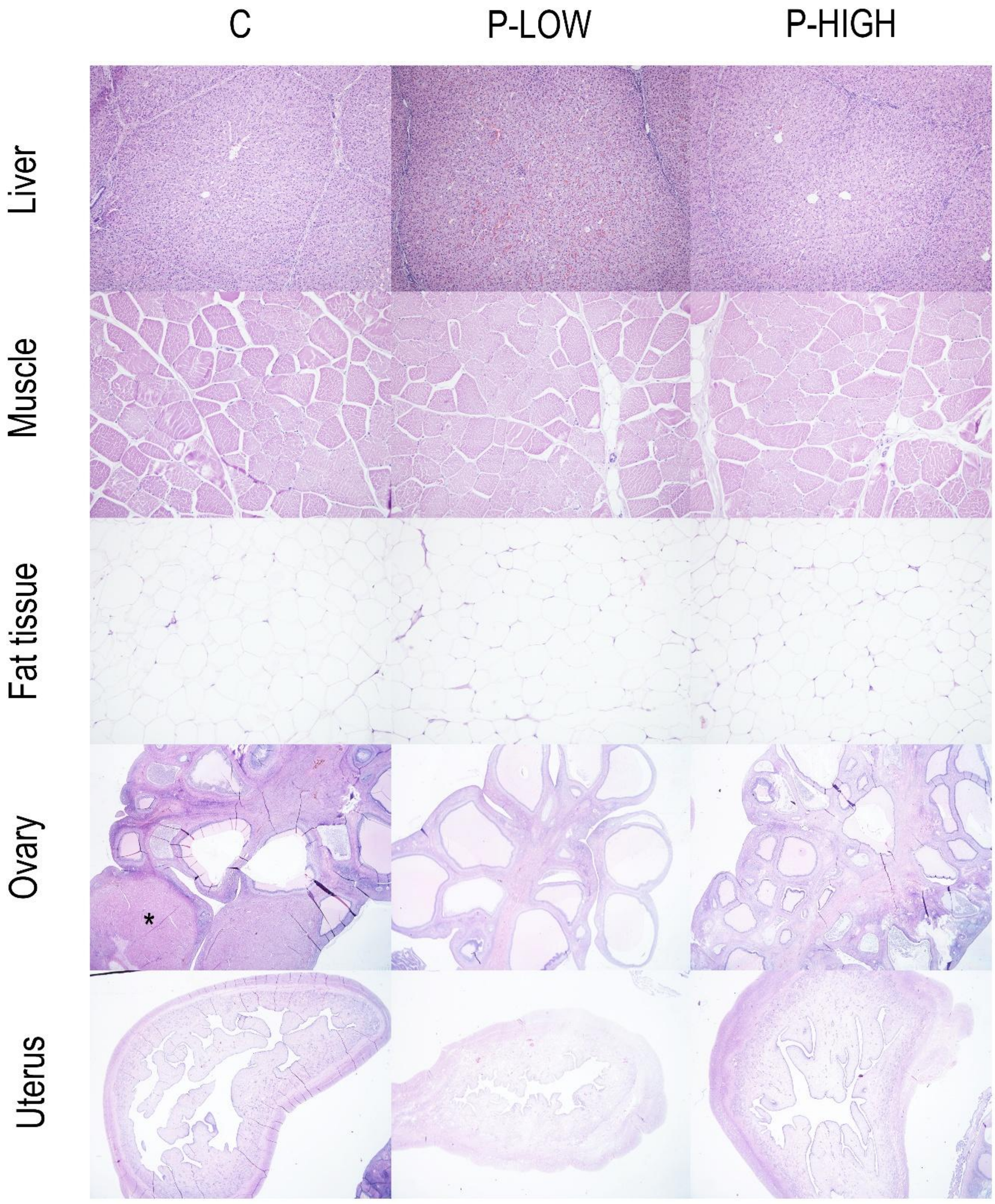

Supplemental Figure 1. Representative histological images of liver, muscle, adipose tissue, ovary, and uterus from the three experimental groups (C, Control; P-LOW; P-HIGH). The liver shows preserved tissue architecture with no evident pathological changes in all groups (Hematoxylin and Eosin, ×10 objective). Skeletal muscle shows normal morphology without evident pathological changes (Hematoxylin and Eosin, ×20 objective). Adipose tissue shows normal architecture with predominantly largely vacuolated adipocytes and absence of pathological changes across all groups (Hematoxylin and Eosin, ×20 objective). Ovary presents preserved morphology with variable presence of developing follicles at different stages and corpora lutea (asterisk) (Hematoxylin and Eosin, ×1.25 objective). Uterus shows normal histological architecture with no detectable pathological changes in any experimental group (Hematoxylin and Eosin, ×1.25 objective).
